# Imagining the Future to Reshape the Past: A Path to Combine Cue Extinction and Memory Reconsolidation With Episodic Foresight for Addiction Treatment

**DOI:** 10.3389/fpsyt.2021.692645

**Published:** 2021-07-21

**Authors:** Parnian Rafei, Tara Rezapour, Warren K. Bickel, Hamed Ekhtiari

**Affiliations:** ^1^Department of Psychology, Faculty of Psychology and Education, University of Tehran, Tehran, Iran; ^2^Iranian National Center for Addiction Studies, Tehran University of Medical Sciences, Tehran, Iran; ^3^Department of Cognitive Psychology, Institute of Cognitive Sciences Studies, Tehran, Iran; ^4^Addiction Recovery Research Center, Center for Transformative Research on Health Behaviors, Fralin Biomedical Research Institute at Virginia Tech Carilion, Roanoke, VA, United States; ^5^Laureate Institute for Brain Research, Tulsa, OK, United States

**Keywords:** cue-exposure therapy, episodic future thinking, addiction, memory reconsolidation, cognitive training, episodic foresight

## Introduction

Continuous maladaptive drug-related memories that are resistant to extinction and cause drug-seeking behaviors to be triggered are known to be one of the hallmarks of drug addiction (1). These drug-related memories are salient, strong, and persistent due to chronic maladaptive consolidation processes. Due to the salient content of drug-related memories formed during drug-taking behaviors, certain stimuli (e.g., peers, locations, paraphernalia) become encoded with reward contingencies associated with drugs. As a result of this learning processing, drug-paired stimuli acquire incentive motivational properties that change them into salient cues (2). According to Pavlovian conditioning, consequent exposure to these stimuli (*Henceforth called* drug cues) activates the original memories and evokes craving. This enhanced retrieval co-occurs with the activation of limbic cortico-striatal pathways involved in reward processing (3). A serious question in addiction neuroscience is whether these memories could be actively erased/reshaped in favor of the recovery process. Different research groups suggested various treatment strategies during the last decade to modulate these memories. Here in this short opinion paper, we propose a novel framework titled “Cue-induced Retrieval and Reconsolidation with Episodic Foresight” (CIREF) that aims to combine three different cognitive interventions, i.e., cue-exposure, memory reconsolidation, and episodic future thinking, to reshape these maladaptive drug-related memories toward more adaptive memories to support addiction recovery.

## Memory Reconsolidation and Cue Exposure

Several studies revealed that when old consolidated memories are reactivated, they may become transiently liable to change their content and salient features (4, 5). This reconsolidation stage that lasts between 1 and 6 h, followed by memory reactivation (6), provides a critical time window to reduce memories' motivational and emotional salience. This “destabilization process” necessitates a subsequent period of restabilization, or *memory reconsolidation*, during which the reactivated memory could be updated, strengthened, modified, disrupted, or erased (7, 8). Some addiction researchers and clinicians hope that renewal of the drug-related memories and alteration of their related motivational and emotional salience via memory reconsolidation could reduce the risk of relapse (9).

To begin the drug-related memory reconsolidation process, patients could be presented with drug cues that make them mentally travel back in time and retrieve the emotional experiences for further elaboration. Previously conducted trials that implemented behavioral memory reconsolidation interventions were often supplemented by extinction-enhancing pharmacological treatments such as D-cycloserine (10–12) or β-adrenergic antagonists like Propranolol (13, 14) and have shown to be impactful in terms of drug cue reactivity extinction (15–17). Positive results in the extinction of drug cue reactivity are suggested to be highly dependent on the efficiency of reactivating maladaptive drug memories (18, 19), and that comes as no surprise that drug cue-exposure seems like a promising strategy for aiding retrieval and reactivation of such memories.

In the experimental context of addiction research, *drug cue-exposure*, in which the drug-related cues are repeatedly presented in the absence of reinforcement (20), is supposed to reduce drug saliency cues. In the clinical context, Cue-Exposure Therapies (CET) for addiction recovery have been developed to extinguish the conditioned craving-provoking effects of drug cues using extinction procedures (21).

At the same time, despite the promising findings of experimental trials that utilized CET and memory reconsolidation paradigms for addiction treatment, mixed results have been obtained regarding the efficacy of these approaches in terms of craving, relapse rate, and the number of abstinent days in the actual treatment setting (22–24). More recently, skeptical arguments have questioned the efficacy as well as the assumption underlying CET trials—which is drug cue extinction in the lab settings could be translated to a reduction of cue-reactivity in real-life settings, leading to lessened problematic drug use—questioning the efficacy and the ecological validity of CET for drug addiction (25). Moreover, a guided approach that leads to opting for healthier “alternatives” is lacking through learning to react to drug-cues in a neutral way (i.e., CET's ideal outcome) and modifying persistent maladaptive drug-related memories (i.e., memory reconsolidation's ideal outcome). Therefore, this shortcoming raises the need for a rigorous, multi-faceted add-on to these approaches to target not only the past and present-oriented aspects of cognitive processing of drug-related memories but also implement a tool for choosing healthier alternatives in response to drug-cues and the reactivation of drug-related memories. Similarly, given the key contribution of future decision-making in four main phases of addiction (*initiation, progression, treatment-seeking*, and *recovery*), interventions that effectively target aberrant decision-making and ultimately effectuate foreseeing the steps leading to recovery are crucial additions to reshape those aspects of cognitive processing modified in aid of CET and memory reconsolidation prospectively (26).

In this opinion paper and for the first time, we propose a new framework as a cognitive intervention termed Cue-induced Retrieval and Reconsolidation with Episodic Foresight (CIREF) for utilizing a combination of episodic future thinking with cue-induced memory reconsolidation to confer greater benefits by adding a future-oriented cognitive training modality.

## Episodic Future Thinking: How Could it Put Extinction into Practice?

Despite the widely conceived notion about memory and its retrospective nature over decades ago, cognitive psychologists and neuroscientists' attention has been recently drawn to the future-oriented aspect of memory. This heed was majorly inspired by Tulving's conception of episodic memory and mental time travel, highlighting the prospective facet of human memory (27, 28). Future thinking or prospection (29) has four primary steps: *simulation, prediction, intention*, and *planning*; 28), which provide the capacity to imagine and project oneself forward in time and to pre-experience personal events that might happen in the future (30–32).

EFT has become a focus of growing interest among neuroscientists and psychologists, most probably owing to its vast contributions to various cognitive functions and adaptive behaviors, such as decision-making, planning, self-control, goal-attainment, goal-directed behavior, and psychological well-being in general (33–36). Moreover, EFT has considerable implications in “implementation intentions” as a deliberate self-regulatory strategy. Pre-deciding how to implement one's goals, simulating the mental representations of probable future events related to a specific goal, and specifying the fully detailed steps leading to goal attainment take place with the aid of EFT (30, 37). Hence, the ability to elaborately simulate possible future events stands as an essential factor in the treatment of mental health issues such as addictive behaviors, given their associations with impaired value-based decision-making and goal-directed behaviors.

EFT has been recently utilized as an intervention in both clinical and non-clinical populations (38). This dynamism mainly results from evidence showing the adaptive function of the EFT, allowing individuals to simulate distant outcomes and desires (39). In other words, the ability to envision future events may result in more accurate predictions of future behaviors and outcomes by allowing one to mentally “try” various potential ways to react to upcoming situations without engaging in actual behaviors (40). Across different populations, EFT has been shown to enhance the prospective memory—remembering to do something in the future at a specific time, which comprises planning, coordinating, and executing one's intention in an appropriate time in the future; for instance, remembering to take a medicine at a specific time of the day (e.g., tomorrow at 10 a.m.) (41–45). Studies suggest that individuals with drug use or other addictive behaviors experience difficulties with prospective memory that could reduce their ability to form a memory-dependent strategy, such as forming the intention and plans to quit drug use. Hence, cognitive training interventions that target prospective memory in the context of drug addiction could be effectively implemented by rehearsing the simulation and planning self-initiated strategies within probable risky situations to achieve intention completion and control drug-seeking behavior in these populations (46).

Another cognitive mechanism that EFT has effectively targeted in several cognitive enhancement studies in samples with addictive behaviors is intertemporal value-based decision-making—choosing between options associated with rewarding outcomes at different time points in the future (47). Numerous theories have proposed that the discounting of delayed rewards with a preference for immediate payoffs compared to greater but delayed ones (i.e., delay discounting) is impaired decision-making that contributes to the development of addictive behaviors [e.g., (48–51)]. Peters and Büchel were the first to show that engaging in EFT reduces delay discounting rates by modulating decision-making and EFT neural networks (including the anterior cingulate cortex, hippocampus, and amygdala). They further showed that these networks enable future-minded choices allowing one to opt for options that maximize future payoffs (52). Moreover, these critical insights contributed to the formation of Reinforcer Pathology Theory (RPT) (53, 54).

Simply put, RPT states that reinforcers are integrated over a temporal window, measured by delay discounting. The length of that window in part determines the relative reinforcing value of substances vs. the other positive pro-social events. Importantly, this perspective recognizes the important temporal features of these different reinforcers. Drugs are brief, immediate, intense, and reliable. At the same time, pro-social reinforcers are less intense, variable in their outcome (e.g., good, bad, or neutral day at work), and that value accrues over time and investment. When the temporal window is short, brief, intense, reliable reinforcers would have greater value. In contrast, a longer temporal window will decrease substance valuation and increase the valuation of pro-social reinforcers.

In light of these advances and seminal findings, several experimental studies and clinical trials investigated the therapeutic effects of EFT on reducing delay discounting and consequent maladaptive behaviors and reported positive health-related outcomes as a result of engaging in EFT in people with alcohol use disorder, overweight, obese and prediabetic individuals, cigarette smokers, cannabis users, and people with cocaine use disorder (55–61). Moreover, EFT training for individuals with addictive behaviors is suggested to improve the efficiency of other psychosocial interventions aiming to attain emotional reappraisal and correction (62). Lastly, the repeated regeneration of episodic future thinking events has been shown to progressively increase the temporal window in those with alcohol use disorder (63). Since addictive behaviors are primarily associated with the pervasive preference of smaller immediate rewards in lieu of larger delayed ones (i.e., steep discounting), and this preference often leads to impulsive maladaptive behaviors such as drug-seeking and drug use (64), the therapeutic effects of EFT potentially arise from its ability to reduce discounting rates. The studies that implemented EFT as an intervention suggested that pre-experiencing future actions broadens one's temporal window by simulating the value of the reward and therefore facilitating the evaluation of behavior's long-term outcomes (e.g., becoming overweight resulting from excessive calorie intake, developing lung cancer resulting from smoking) (65). These findings indicate that EFT has therapeutic effects on addictive behaviors by changing the excessive discounting of the future while promoting healthy and adaptive decisions resulting in positive behavior change. Considering the aforementioned positive effects, the current paper proposes a new framework for integrating EFT with cue-induced memory reconsolidation in the context of addiction treatment.

## Episodic Future Thinking in Cue Exposure Context

As we discussed before, drug-related memories could be retrieved and reactivated as a result of drug cue-exposure. During this context, patients could be asked to imagine themselves in a hypothetical drug-related situation associated with the presented cue (e.g., being offered to use drugs, passing by a group of drug-users in a park, etc.) taking place in the future and elaborate on it in episodic details. The five stages of the CIREF intervention take place in the same order as the EFT stages and subsequent to the cue-exposure as follows:

**1) Activating Past Memories With Cue Exposure:** Patients initially become exposed to drug-related stimuli using formerly validated pictorial cues (66). This drug cue-exposure process leads to reactivation of maladaptive drug-related memories that happened in the past, which causes the patients to retrieve the drug-related memories and possibly re-experience the emotional arousal associated with them. Past memories become unstable during the reactivation stage and become ready to undergo potential modifications throughout the next stages.
**2) Simulating Future Cue Exposures:** Patients are prompted to vividly simulate a novel future event that may happen in response to drug-cue encounter in as much detail as possible and verbally describe who they are with, what they are doing (and thinking), where they exactly are, and how they feel (*Simulation* phase of EFT). Imagining the probable future events in the proposed manner would improve the ecological validity of the intervention.
**3) Predicting Response to Cue Exposure and Its Outcomes:** Subsequently, patients are asked to predict their associated emotions and behaviors in the simulated event (*Prediction* phase of EFT). As the patients verbally express their predictions, different options of how to deal with the potential drug-related situation should be predicted and vividly imagined. During this stage, patients expect both positive and negative scenarios that may happen due to being exposed to drug-related situations. The probable future behaviors and emotions (both positive and negative) undergo a *pre-appraisal* stage by the patient based on the predicted outcomes.
**4) Making Intentions in the Context of Cue Exposure:** Upon prediction of their reactions, patients are guided to replace immediate rewards that may be chosen impulsively with later self-controlled reward choices (*Intention* phase of EFT). The intention formation phase in this framework is similar to the “goal-setting” exercises taking place in psychotherapy settings and implementation of intentions (37) in which the patient specifies the *when, where, and how* of responses leading to goal attainment.
**5) Developing Executive Plan for Adaptive Response:** Finally, the unstable retrieved drug-related memory, therefore, will be updated with memory reconsolidation strategies that are not limited to modification of the retrospective memory *per se* but also supplemented with the reconstruction of the prospective memory leading to optimal planning for the future and behaving upon it (*Planning* phase of EFT). During the planning phase, patients are guided to plan the organization of steps needed to arrive at a specific autobiographical future outcome (67).

**Figure 1:**
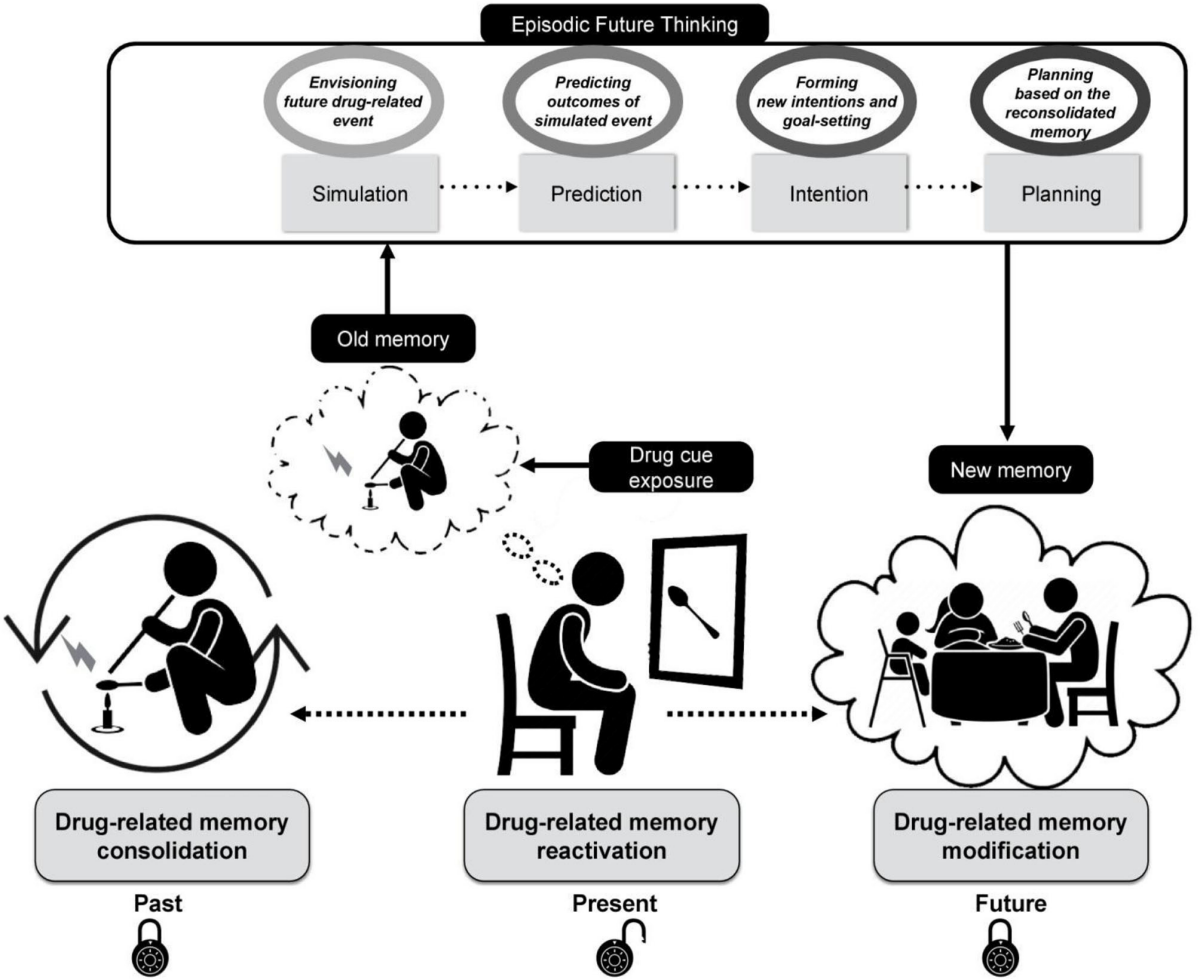
A schematic diagram illustrating the cognitive processes underlying “Cue-induced Retrieval and Reconsolidation with Episodic Foresight (CIREF),” which applies episodic future thinking to cue-induced memory reconsolidation. The maladaptive drug-related memory that has been consolidated and stored in the past (Left) becomes reactivated as a result of drug cue exposure (Middle). The reactivated memory becomes transiently labile and prone to modification. The extinction process of maladaptive drug-related memories takes place as a result of repetitive exposure to relevant drug cues. Implementing episodic future thinking during this stage engages the person in (1) simulation of a future drug-related event, (2) prediction of emotions and reactions related to it, (3) intention of modifying the drug-seeking behavior triggered by cue exposure, and (4) planning for future actions in response to drug cue exposure maintaining healthy behavior (Top). The boxes in oval shapes depict processes underlying each stage. The reconsolidated memory is therefore updated and re-encoded in aid of episodic future thinking, and the drug cue has undergone extinction as a result of cue exposure therapy (Right).

To put it differently, while the patients undergo the CIREF cognitive intervention (multiple sessions of individual or group-based therapy meetings), the maladaptive drug-related memories become triggered by a stepwise exposure using a large database of drug-related stimuli. After exposure to each individually validated drug cue set, the patients are asked to imagine themselves in a hypothetical cue-associated drug-related situation that could be happening in the future and elaborate on it in episodic details, mentally predicting and “trying out” different options and their outcomes and planning their future actions upon them. Then, the planned activities based on reconsolidated memories become stored as a new prospective memory guiding the patients to recall their planned intentions at some future point in time. Figure 1 illustrates the conceptual process of the proposed framework and the implications of each stage of this approach in real-life settings.

CIREF benefits from EFT enhancement as a translatable approach in clinical settings (68) that is also necessitated by pieces of evidence showing that individuals with addictive behaviors have difficulties imagining future events and implementing intentions based on them (69–72). Moreover, the suggested framework could fill in the gaps of CET and memory reconsolidation interventions by taking a step further from classical conditioning and updating past drug-related memories by implementing goal-based strategies. Individuals struggling with addiction could develop their “future sightedness” and increase the length of their temporal window trained via EFT within this framework and consequently make healthier decisions, possibly by viewing future events as more connected to their present (73).

## Future Directions and Conclusion

Theoretically, two sets of clinical outcomes are expected to be accomplished at both neural and behavioral levels after individuals with addictive behaviors undergo the CIREF intervention. The first set of which are short-term outcomes comprising cue reactivity—the physiological and subjective reactions while being exposed to drug-related stimuli—and drug craving (i.e., feeling the urge to use drugs or be engaged with addictive behavior). These outcomes are expected to be immediate changes in patients' behavior after completion of the CIREF intervention and could be measured with self-report measures (e.g., craving scales and questionnaires), as well as brain imaging techniques (e.g., cue reactivity fMRI task) (74). Ideally, we are expecting that the CIREF approach would lead to some long-term outcomes as well. The long-term clinical outcomes include changes in abstinence measures, such as duration of abstinence (usually measured by biochemical validation methods like urine drug tests in the context of substance use disorders), type of abstinence (i.e., point prevalence, continuous, or prolonged), and relapse rates (75). Therefore, the clinical outcomes of the CIREF approach could be validated at multiple levels using measurements of the neural and cognitive targets (as mediators) and ultimate behavioral outcomes in future studies.

There are potential limitations to the CIREF approach. For instance, the person who is guiding the intervention (i.e., the therapist) has to be conscious of the cue-induced craving levels and ensure that the patients' cue-reactivity and craving that are triggered by the drug cue encounter and simulation (step 1 and 2 of the CIREF intervention) will be managed and mitigated effectively before the patient starts to form intentions and plan for healthier outcomes (step 4 and 5 of the CIREF intervention). A self-report assessment of craving before and after each session of the intervention and ending the session with common psychological craving management strategies (76) could potentially address this limitation as it helps the therapist to gain more control over patients' cue-elicited craving.

Furthermore, there is a thorough and in-depth protocol paper in preparation by our team of authors elaborating on each stage of the CIREF framework that provides the detailed considerations that should be taken into account while implementing each stage of this multicomponent intervention and its translational limitations.

In sum, addiction is a complex disorder that may persist due to a lack of proper integration of past memories and new learning. We propose a novel cognitive interventional framework for drug addiction titled “Cue-Induced Retrieval and Reconsolidation with Episodic Foresight (CIREF),” aiming to supplement cue-induced memory reconsolidation strategies focused on retrieval-extinction procedures with episodic future thinking for optimal results. Episodic future thinking guides patients with addictive behaviors to simulate future events that trigger cue-induced drug craving and mentally rehearse coping strategies that lead to addiction recovery. CIREF provides a multi-faceted approach for addiction treatment in light of targeting both past and future-oriented cognition affected by addiction. Further research is needed to bridge the gap between fundamental laboratory research and applied research to translate the presented framework's basic idea into an actual manualized or computerized intervention for future clinical investigations.

## Author Contributions

PR, TR, and HE conceived the conceptual framework of the paper. PR and TR wrote the first draft of the manuscript. HE and WB edited the manuscript and gave conceptual advice. All authors discussed the implications and commented on the final manuscript.

## Conflict of Interest

The authors declare that the research was conducted in the absence of any commercial or financial relationships that could be construed as a potential conflict of interest.

## Figures and Tables

**Table:** 

Cue-exposure therapy	A behavior therapy technique in which a patient is exposed to stimuli that induce cravings for specific substances of use (e.g., alcohol) while the therapist uses other techniques to reduce or eliminate the craving and prevent their habitual response (i.e., drug use) (77, 78).
Memory reconsolidation	The active process of restabilizng a reactivated memory that has been formerly stored in long-term memory, while new information is incorporated during reconsolidation into an updated memory (8, 79).
Episodic future thinking	The mental capacity to imagine or simulate events or experiences that might occur in one's personal future to pre-experience a probable event (30, 31).
Reinforcer pathology theory	The Reinforcer Pathology theory proposes conditions that result in an excessive valuation of addictive substances/behaviors as observed in addictive disorders (e.g., drug addiction, overeating). This approach identifies and measures a process that is well-correlated with a disorder or disease, followed by interventions designed to change that disease-correlated process and assess its effects on other aspects of the disorder (53, 80).
Incentive salience theory	The Incentive Salience Theory of addiction suggests that addiction is caused primarily by drug-induced sensitization in the brain mesocorticolimbic pathways that attribute incentive salience to reward-associated stimuli. This theory proposes that sensitization of the neural systems responsible for incentive salience (drug wanting) can occur independently of changes in neural systems that mediate the subjective pleasurable effects of drugs (drug “liking”) (81, 82).
Delay discounting	The decrease in the present subjective value of a reward as the delay to its receipt increases. Delay discounting is a commonly used behavioral measure of impulsive decision making (83, 84).
Implementation intentions	Implementation intentions are if-then plans specifying when, where, and how the person will set their actions into motion that spell out in advance how one wants to reach a goal (85).
